# Author Correction: Local genetic correlations exist among neurodegenerative and neuropsychiatric diseases

**DOI:** 10.1038/s41531-023-00537-6

**Published:** 2023-05-30

**Authors:** Regina H. Reynolds, Aaron Z. Wagen, Frida Lona-Durazo, Sonja W. Scholz, Maryam Shoai, John Hardy, Sarah A. Gagliano Taliun, Mina Ryten

**Affiliations:** 1 Genetics and Genomic Medicine, Great Ormond Street Institute of Child Health, University College London, London, UK; 2 Aligning Science Across Parkinson’s (ASAP) Collaborative Research Network, Chevy Chase, MD USA; 3 Department of Clinical and Movement Neurosciences, Queen Square Institute of Neurology, London, UK; 4 Neurodegeneration Biology Laboratory, The Francis Crick Institute, London, UK; 5 Montréal Heart Institute, Montréal, QC Canada; 6 Faculty of Medicine, Université de Montréal, Montréal, Canada; 7 Neurodegenerative Diseases Research Unit, National Institute of Neurological Disorders and Stroke, Bethesda, MD USA; 8 Department of Neurology, Johns Hopkins University Medical Center, Baltimore, MD USA; 9 Department of Neurodegenerative Diseases, Queen Square Institute of Neurology, University College London, London, UK; 10 UK Dementia Research Institute, University College London, London, UK; 11 Department of Medicine & Department of Neurosciences, Université de Montréal, Montréal, QC Canada; 12 NIHR Great Ormond Street Hospital Biomedical Research Centre, University College London, London, UK

**Keywords:** Genomics, Neurodegenerative diseases

Correction to: *npj Parkinson’s Disease* 10.1038/s41531-023-00504-1, published online 28 April 2023

In this article the affiliation ‘Aligning Science Across Parkinson’s (ASAP) Collaborative Research Network, Chevy Chase, MD, USA’ for Author Maryam Shoai was missing. The original article has been corrected.

