# Supplementary material for: Local genetic correlations exist among neurodegenerative and neuropsychiatric diseases
Source: NPJ Parkinsons Dis. 2023 Apr 28;9:70. doi: 10.1038/s41531-023-00504-1 (PMC10147945; doi:10.1038/s41531-023-00504-1)
Supplement: Supplementary file 1 — Supplementary Information [file 41531_2023_504_MOESM1_ESM.pdf]

Supplementary Information

Local genetic correlations exist among neurodegenerative and neuropsychiatric diseases

Regina H. Reynolds, Aaron Z. Wagen, Frida Lona-Durazo, Sonja W. Scholz, Maryam Shoai, John Hardy, Sarah A. Gagliano Taliun, Mina Ryten

Contents

Supplementary Figures 2

Supplementary Tables 11

Supplementary Note 12

Comparison of local r_gs between disease traits to existing reports of local genetic relations 12

Sensitivity analysis indicates that by-proxy cases do not drive spurious local correlations among neurodegenerative diseases 13

Extended discussion of results of local multiple regression.. 14

One significant predictor trait in a two-predictor model 14

Non-significant predictor traits 14

References 16

Supplementary Figures

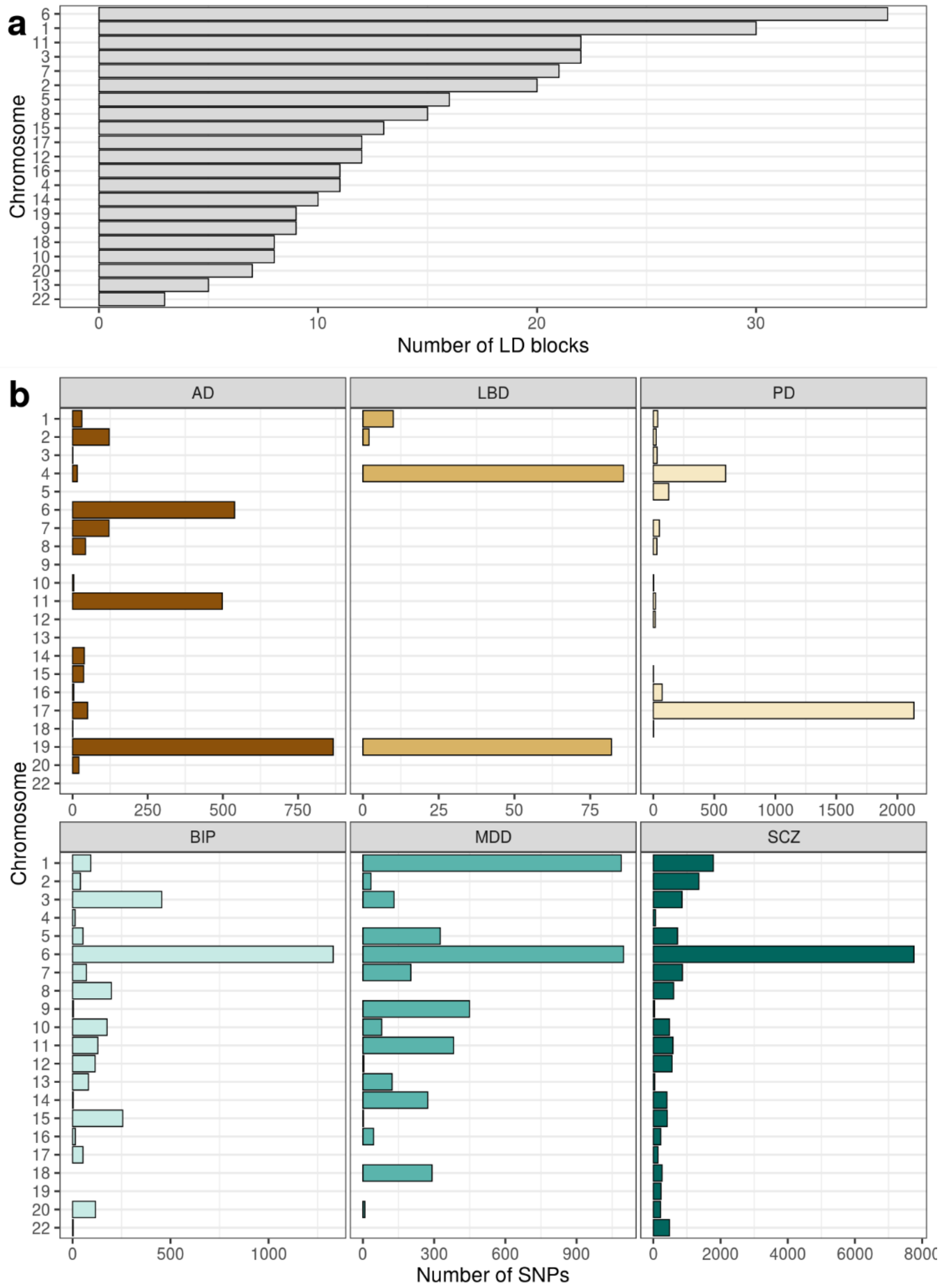

Supplementary Figure 1 (a) Number of LD blocks containing genome-wide significant loci per chromosome. Chromosomes have been ordered by the total number of LD blocks in each chromosome. **(b)** Number of genome-wide significant AD, BIP, LBD, MDD, PD and SCZ SNPs per autosome.

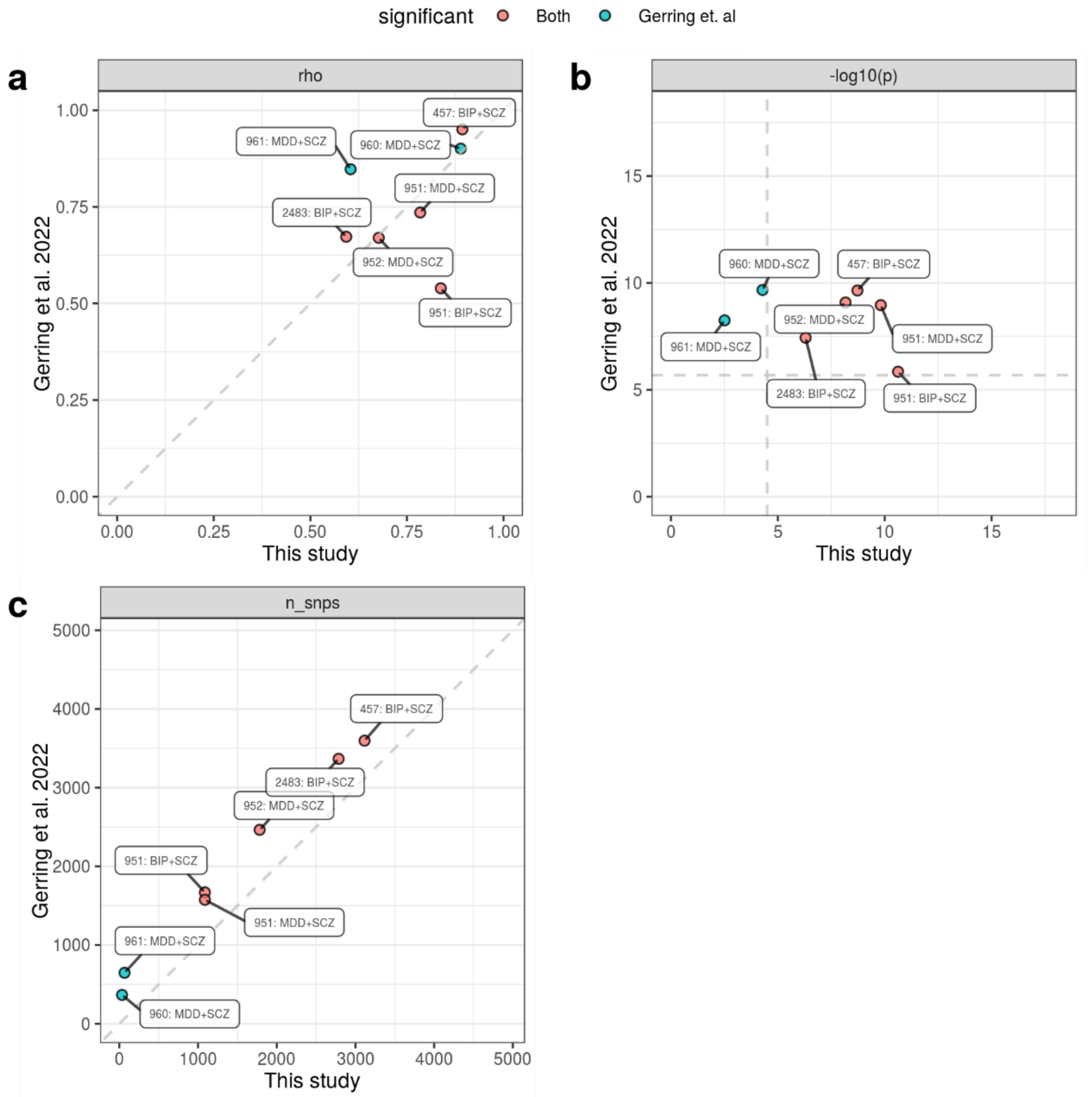

Supplementary Figure 2 Scatter plot of **(a)** the standardised coefficient for r_g (rho, ρ), **(b)** -log10(p-value) and **(c)** number of overlapping SNPs for each pair of traits that overlapped between our study and the study by Gerring *et al.*[1] In **(a)** the dashed lines indicate significance thresholds in each study (Gerring *et al.*, p < 0.05/24,054; this study, p < 0.05/1603) and in **(b, c)** the dashed line represents the line y = x. Points in both plots are coloured by whether they passed significance thresholds in both studies or only the Gerring *et al.* study. Points are labelled by LD block and trait pair. Coordinates for LD blocks (in the format chromosome:start-end, GRCh37): 457, chr3:36,840,137-38,729,767; 951, chr6:26,396,201-27,261,035; 952, chr6:27,261,036-28,666,364; 960, chr6: 31,320,269-31,427,209; 961, chr6:31,427,210-32,208,901; 2483, chr22:38,718,590-40,378,783 .

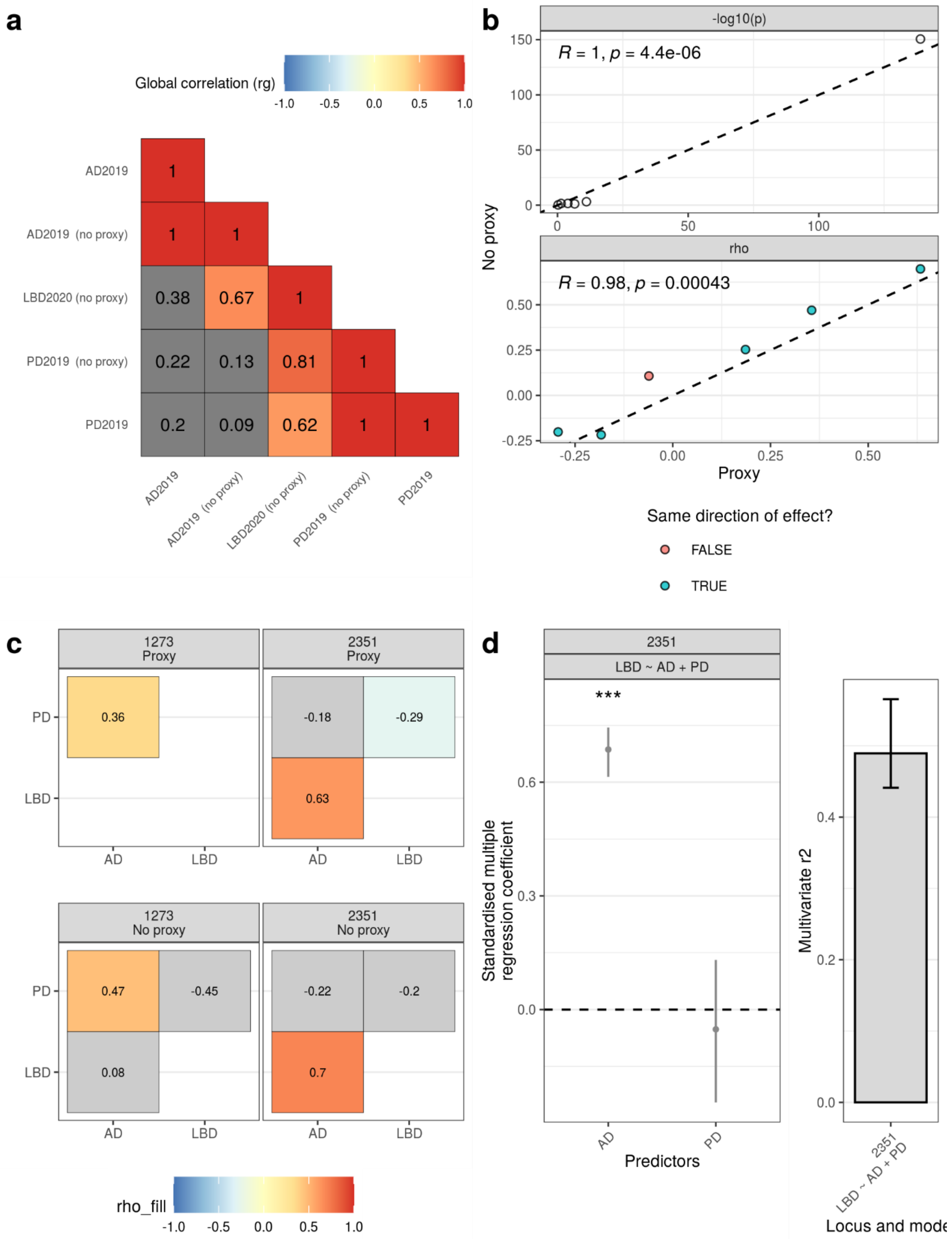

Supplementary Figure 3 Impact of excluding UK Biobank by-proxy cases on global genetic correlations, local genetic correlations and multiple regression.

(a) Global genetic correlations between pairs of phenotypes. Significant negative and positive correlations are indicated by blue and red fill, respectively. Non-significant correlations (p >= 0.05/n_tests) have a grey fill. **(b)** Scatter plot of -log10(p-value) and the standardised coefficient for r_g (rho, ρ) for each pair of traits with sufficient univariate signal to carry out a bivariate test using AD/PD GWASs with or without by-proxy cases. In each panel, Pearson's coefficient (R)

and associated p-value (p) are displayed. The black dashed line represents the line y = x. Points are coloured, where applicable, by whether they share the same direction of effect. **(c)** Significant bivariate local genetic correlations using AD/PD GWASs with or without by-proxy cases (as indicated in panel headers). Heatmaps show the rho for all tested associations within the LD block, with significant negative and positive correlations indicated by blue and red fill, respectively. Non-significant correlations have a grey fill. **(d)** Results of multiple regression model across LD block 2351. Plot (left) of standardised coefficients for each predictor in multiple regression model in LD block 2351, with whiskers spanning the 95% confidence interval for the coefficients. Plot (right) of multivariate r^2 for LD block 2351, where multivariate r^2 represents the proportion of variance in genetic signal for LBD explained by AD and PD simultaneously. Whiskers span the 95% confidence interval for the multivariate r^2. ***, p < 0.001. Coordinates for LD blocks (in the format chromosome:start-end, GRCh37): 1273, chr8:27,406,512-28,344,176; 2351, chr19:45,040,933-45,893,307.

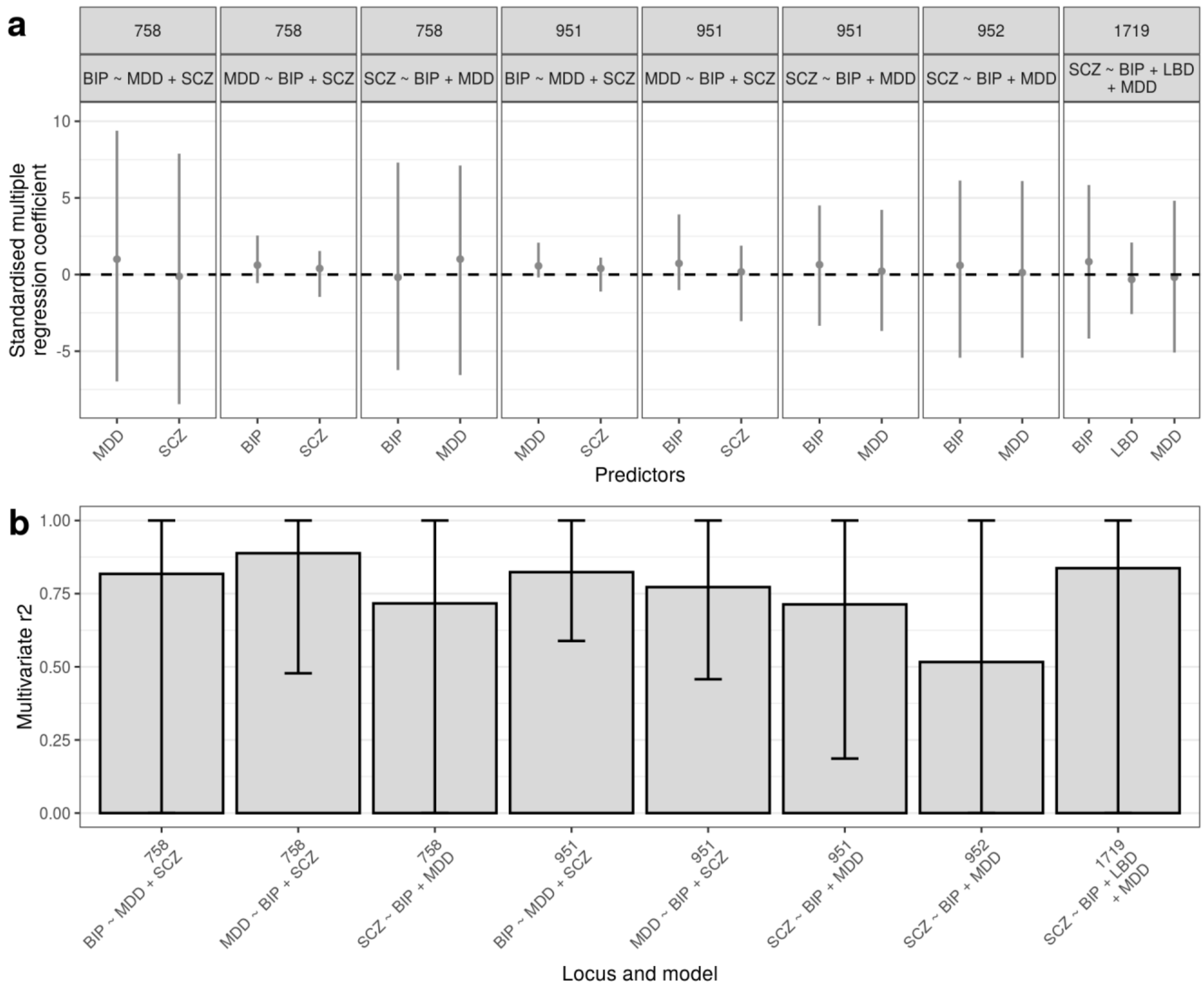

Supplementary Figure 4 Multiple regression across LD blocks with multiple trait pair correlations.

For both plots, only those multiple regression models with no significant predictors ($p < 0.05$) are shown. **(a)** Plots of standardised coefficients for each predictor in multiple regression models across each LD block, with whiskers spanning the 95% confidence interval for the coefficients. Panels are labelled by the LD block identifier and the regression model. **(b)** Multivariate r^2 for each LD block and model, where multivariate r^2 represents the proportion of variance in genetic signal for the outcome trait explained by all predictor traits simultaneously. Whiskers span the 95% confidence interval for the r^2. Coordinates for LD blocks (in the format chromosome:start-end, GRCh37): 758, chr4:175,959,696-177,129,678; 951, chr6:26,396,203-27,261,035; 952, chr6:27,261,036-28,666,364; 1719, chr11:112,755,447-113,889,019.

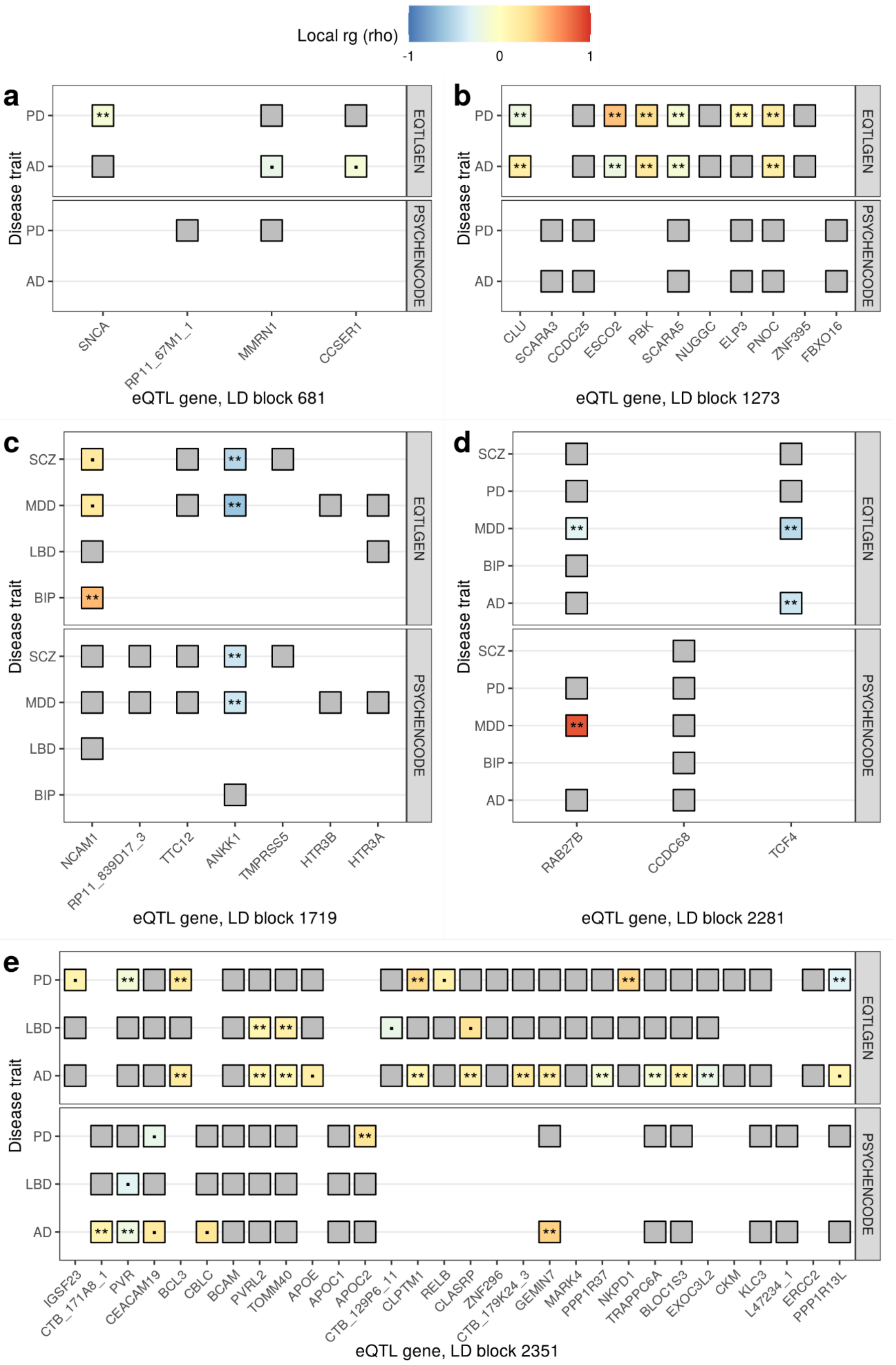

Supplementary Figure 5 Local gene expression and disease trait correlations across 5 LD blocks of interest.

Heatmaps of the standardised coefficient for r_g (rho) for all tested gene expression-disease trait correlation within LD block **(a)** 681, **(b)** 1273, **(c)** 1719, **(d)** 2281 and **(e)** 2351. All negative and positive r_gs with p < 0.05 are indicated by blue

and red colour, respectively, while the remainder have a grey fill. Significant local r_gs (FDR < 0.05) are indicated by two asterisks (**), while nominally significant local r_gs ($p < 0.05$) are indicated with a black square (▪). Genes are ordered left to right on the x-axis by the genomic coordinate of their gene start. Coordinates for LD blocks (in the format chromosome:start-end, GRCh37): 681, chr4:90,236,972-91,309,863; 1273, chr8:27,406,512-28,344,176; 1719, chr11:112,755,447-113,889,019; 2281, chr18:52,512,524-53,762,996; 2351, chr19:45,040,933-45,893,307.

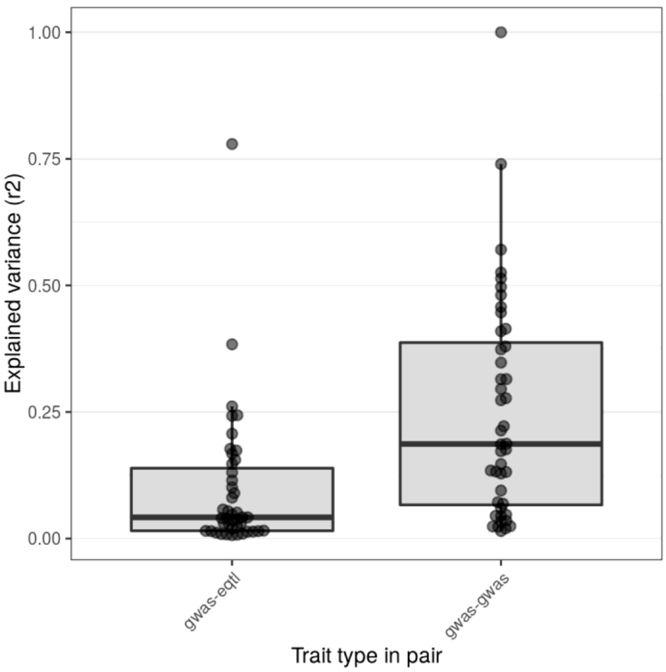

Supplementary Figure 6 Explained variance in trait pairs with different trait types.

Boxplot of explained variance (r^2, the proportion of variance in genetic signal of one disease trait in a pair explained by the other) in trait pairs involving a disease and gene expression trait (gwas-eqtl) or two disease traits (gwas-gwas). Only local r_gs that passed significance are plotted (FDR < 0.05; N, local r_gs = 87).

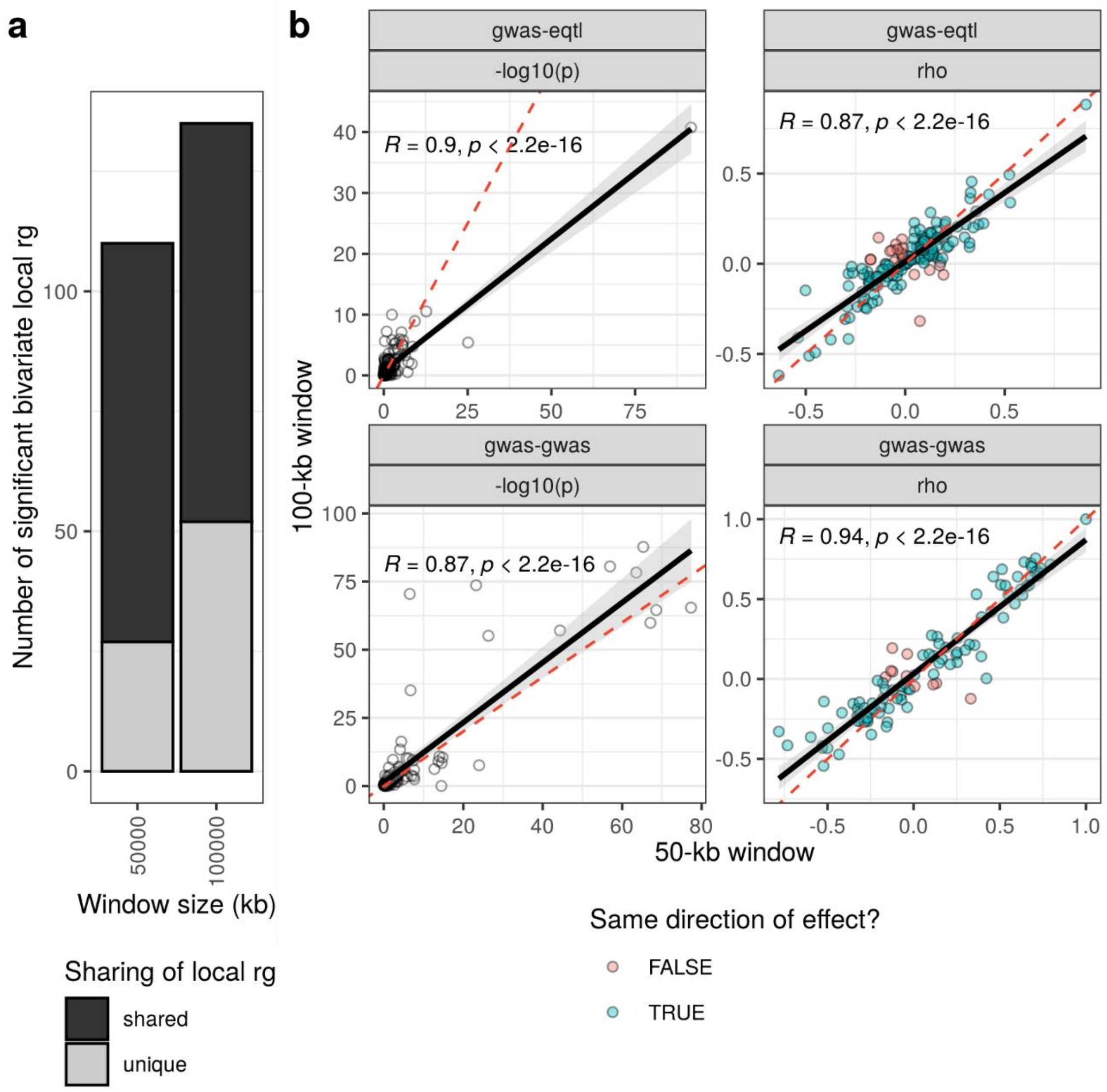

Supplementary Figure 7 Effect of window size on local genetic correlations.
(a) Number of significant bivariate local r_g's across window sizes. Bars are coloured by whether r_g's are significant across both window sizes (shared) or only one (unique). (b) Scatter plot of -log10(p-value) and the standardised coefficient for r_g (rho, ρ) for each pair of traits that could be tested across genic regions with a 50-kb or 100-kb window. Panels indicate whether the pair of traits included a disease and gene expression trait (gwas-eqtl) or two disease traits (gwas-gwas). Points are coloured by whether they share the same direction of effect. The black line represents a linear model fitted to the data, with the 99% confidence interval indicated with a grey fill. Further, Pearson's coefficient (R) and associated p-value (p) are displayed. The red dashed line represents the line y = x.

Supplementary Tables

Supplementary Table 1 LD blocks, their associated disease traits (as determined by overlap of genome-wide significant SNPs) and overlapping genes.

Supplementary Table 2 Results of LDSC using the six disease traits.

Supplementary Table 3 Results of LAVA using the six disease traits.

Supplementary Table 4 Overlaps between results of LAVA and results from Drange *et al*[2], Smeland *et al.*[3] and Gerring *et al.*[1]

Supplementary Table 5 Results of LAVA using GWASs for AD and PD that exclude UK Biobank by-proxy cases.

Supplementary Table 6 Results of multiple regression analyses.

Supplementary Table 7 Results of LAVA using disease and gene expression traits (100-kb window). Sheets containing bivariate results for each LD block also contain (a) locus plot of genic regions (including 100-kb window). Significant bivariate local genetic correlations between a disease and gene expression trait are highlighted in blue (FDR < 0.05). (b) Edge diagrams for genic regions where a significant bivariate local genetic correlation was observed between a disease and gene expression trait (FDR < 0.05). Edges display the standardised coefficient for genetic correlation (rho) for significant bivariate local genetic correlations, with negative and positive correlations indicated by blue and red colour, respectively. GWAS and eQTL nodes are indicated by grey and white fill, respectively.

Supplementary Table 8 Results of LAVA using disease and gene expression traits (50-kb window). Sheets containing bivariate results for each LD block also contain (a) locus plot of genic regions (including 50-kb window). Significant bivariate local genetic correlations between a disease and gene expression trait are highlighted in blue (FDR < 0.05). (b) Edge diagrams for genic regions where a significant bivariate local genetic correlation was observed between a disease and gene expression trait (FDR < 0.05). Edges display the standardised coefficient for genetic correlation (rho) for significant bivariate local genetic correlations, with negative and positive correlations indicated by blue and red colour, respectively. GWAS and eQTL nodes are indicated by grey and white fill, respectively.

Supplementary Table 9 Results of partial correlations using disease and gene expression traits (100-kb window).

Supplementary Note

Comparison of local r_gs between disease traits to existing reports of local genetic relations

We compared local r_gs to existing results from studies of: (i) AD and PD using rho-HESS[4]; (ii) AD and BIP[5], AD and MDD[6], PD and SCZ[3], all of which used a conditional/conjunctional FDR approach; and (iii) 10 psychiatric disorders and 10 substance abuse phenotypes using LAVA[1]. We were unable to replicate the genetic overlaps between AD and PD reported in the *HLA* (specifically in chr6: 31,571,218-32,682,664[4]) and *MAPT* (rs393152 shown to increase risk of both AD and PD[7]) loci. In the case of the *MAPT* locus, this lack of replication was due to insufficient univariate signal for AD in the LD block containing *MAPT* (LD block 2207, chr17: 43,460,501-44,865,832), while in the *HLA* locus, several of the overlapping LD blocks (LD block 961-6 ranging across chr6: 31,427,210-32,682,213) either had too few overlapping SNPs (i.e. fewer than 2, as in LD block 962, 963 and 966) between the 6 disease traits for adequate signal or negative variance estimates (i.e. PD in LD block 965).

We were unable to replicate any of the associations reported using a conditional/conjunctional FDR approach. For 4 associations (rs11649476 in AD and BIP[5]; rs5011436 in AD and MDD[6]; rs2979160 and rs4921739 in PD and SCZ[3]), this result was attributed to a lack of overlap with the 300 LD blocks tested. For 1 association (rs62333164 in PD and SCZ[3]), this was due to insufficient univariate signal for SCZ in the overlapping LD block (LD block 752, chr4:169555115-170682809). Of the 9 loci that were jointly associated with PD and SCZ[3], 6 intersected with LD blocks where we performed a bivariate test for PD and SCZ, but none of the bivariate tests passed the threshold for significance ($p < 0.05/1,603$; **Supplementary Table 4**, **Supplementary Table 5**).

Of the 14 LD blocks found to contain significant local r_gs between any of BIP, MDD and SCZ in the study by Gerring *et al.,* 6 LD blocks intersected with LD blocks where we performed a bivariate test using the same phenotype pairs. We were able to replicate 5 of the 7 overlapping local r_gs (BIP and SCZ in LD block 457; SCZ and BIP or MDD in LD block 951; MDD and SCZ in LD block 952; and BIP and SCZ in LD block 2483; **Supplementary Figure 2**; **Supplementary Table 4**). Our inability to replicate 2 local r_gs could be due to: (i)

the lower number of overlapping SNPs present in the tested LD blocks in our study compared to Gerring *et al.* (**Supplementary Figure 2c**); and/or (ii) differences in GWASs used (while both studies used the same MDD and SCZ GWASs, our study did not include 23andMe data from the MDD GWAS and used a more recent BIP GWAS).

Sensitivity analysis indicates that by-proxy cases do not drive spurious local correlations among neurodegenerative diseases

Given concerns that UK Biobank (UKBB) by-proxy cases could potentially be mislabelled (i.e. parents of by-proxy case suffered from another type of dementia) and lead to spurious r_gs between neurodegenerative traits, we performed sensitivity analyses using GWASs for AD and PD that excluded UKBB by-proxy cases[8]. Analysis of global r_gs revealed significant positive correlations of 1 between the AD GWASs with and without by-proxy cases and the PD GWASs with and without by-proxy cases (**Supplementary Figure 3**, **Supplementary Table 5**). Of the 21 LD blocks where significant local r_gs were observed among pairs of the 3 neurodegenerative traits using the AD and PD GWASs with by-proxy cases, only 2 LD blocks (LD block 1273, chr8:27,406,512-28,344,176, and LD block 2351, chr19:45,040,933-45,893,307) had sufficient local genetic signal for both AD and PD without by-proxy cases. This limited number of testable LD blocks was expected: reflecting the decrease in cohort numbers when UKBB by-proxy cases are excluded from AD and PD GWASs (**Table 1**). We were able to replicate 2 of the 3 significant local r_gs observed in LD block 1273 (chr8:27,406,512-28,344,176) and 2351 (chr19:45,040,933-45,893,307), including the positive r_g between AD and PD in LD block 1273 and the positive r_g between AD and LBD in LD block 2351 (**Supplementary Figure 3**, **Supplementary Table 5**). Further, while the local r_g between LBD and PD in LD block 2351 was non-significant when using the PD GWAS without by-proxy cases, the correlation was in the same direction in the complementary analysis using the PD GWAS with by-proxy cases (no by-proxy: ρ = -0.201, ρ CI = -0.443 to 0.007, p = 0.061; by-proxy: ρ = -0.293, ρ CI = -0.405 to -0.184, p = 2.75 x 10^{-7}) (**Supplementary Figure 3**, **Supplementary Table 5**).

Extended discussion of results of local multiple regression

This note expands on the results of local multiple regression by providing an example of an LD block where only one predictor trait in a two-predictor model was significant and an example of an LD block where all predictor traits were non-significant.

One significant predictor trait in a two-predictor model

This scenario could indicate that one predictor accounts for the relationship of the outcome trait with other non-significant predictors. This outcome is best illustrated in LD block 952 (chr6:26,396,201-27,261,035), where we observed significant local r_gs between all 3 neuropsychiatric disorders (**Supplementary Table 10**). Notably, only BIP was a significant predictor of MDD when jointly modelled with SCZ, and likewise only MDD was a significant predictor of BIP when jointly modelled with SCZ, and in both cases, the regression coefficient for SCZ was non-significant and substantially lower than the estimated bivariate local r_g. Inclusion of SCZ together with one of BIP or MDD showed little to no improvement in variance in multivariate r^2 over the bivariate r^2 estimated between BIP and MDD, indicating that including SCZ in the model did not improve the explained variance. In other words, BIP likely accounts for the genetic relationship of SCZ with MDD at this locus, while MDD likely accounts for the relationship between SCZ with BIP.

Supplementary Table 10 Bivariate and conditional genetic relations for the 3 neuropsychiatric traits in LD block 952. Parameters refer to either the estimated coefficient for bivariate local r_gs (ρ) or the regression coefficient (γ) from local multiple regression. Coordinates for the LD blocks (in the format chromosome:start-end, GRCh37): chr6:26,396,201-27,261,035.

Method	Model	Predictor	Parameter	R^2	P-value
Bivariate	BIP ~ MDD	-	0.92	0.84	4.92×10^{-9}
Bivariate	BIP ~ SCZ	-	0.72	0.51	8.50×10^{-9}
Bivariate	MDD ~ SCZ	-	0.68	0.46	6.77×10^{-9}
Multiple regression	BIP ~ MDD + SCZ	MDD	0.79	0.86	2.08×10^{-2}
		SCZ	0.18		5.13×10^{-1}
Multiple regression	MDD ~ BIP + SCZ	BIP	0.88	0.84	2.84×10^{-2}
		SCZ	0.04		8.84×10^{-1}
Multiple regression	SCZ ~ BIP + MDD	BIP	0.60	0.52	4.65×10^{-1}
		MDD	0.13		8.61×10^{-1}

Non-significant predictor traits

This scenario could indicate collinearity among trait predictors. For example, in locus 951 (chr6:26,396,201-27,261,035), there were strong bivariate correlations between BIP, MDD and SCZ. Fitting all two-predictor

model combinations among the 3 traits showed only a small increase in multivariate r^2 compared to any of the traits individually, suggesting that all 3 traits essentially capture the same genetic relationship (**Supplementary Table 11**).

Supplementary Table 11 Bivariate and conditional genetic relations for the 3 neuropsychiatric traits in LD block 951. Parameters refer to either the estimated coefficient for bivariate local r_gs (ρ) or the regression coefficient (γ) from local multiple regression. Coordinates for the LD blocks (in the format chromosome:start-end, GRCh37): chr6:26,396,201-27,261,035.

Method	Model	Predictor	Parameter	R^2	P-value
Bivariate	BIP ~ MDD	-	0.87	0.76	2.95×10^{-8}
Bivariate	BIP ~ SCZ	-	0.84	0.70	2.36×10^{-11}
Bivariate	MDD ~ SCZ	-	0.78	0.72	1.52×10^{-10}
Multiple regression	BIP ~ MDD + SCZ	MDD	0.56	0.82	1.57×10^{-1}
		SCZ	0.40		3.11×10^{-1}
Multiple regression	MDD ~ BIP + SCZ	BIP	0.73	0.77	1.92×10^{-1}
		SCZ	0.18		7.11×10^{-1}
Multiple regression	SCZ ~ BIP + MDD	BIP	0.64	0.71	3.56×10^{-1}
		MDD	0.22		7.09×10^{-1}

References

1. Gerring, Z. F., Thorp, J. G., Gamazon, E. R. & Derks, E. M. A Local Genetic Correlation Analysis Provides Biological Insights Into the Shared Genetic Architecture of Psychiatric and Substance Use Phenotypes. *Biol. Psychiatry* **92**, 583–591 (2022).
2. Drange, O. K. *et al.* Genetic overlap between Alzheimer's disease and bipolar disorder implicates the MARK2 and VAC14 genes. *Front. Neurosci.* **13**, 1–11 (2019).
3. Smeland, O. B. *et al.* Genome-wide Association Analysis of Parkinson's Disease and Schizophrenia Reveals Shared Genetic Architecture and Identifies Novel Risk Loci. *Biol. Psychiatry* **89**, 227–235 (2021).
4. Stolp Andersen, M., Tan, M., Holtman, I. R., Hardy, J. & Pihlstrøm, L. Dissecting the limited genetic overlap of Parkinson's and Alzheimer's disease. *Ann. Clin. Transl. Neurol.* 1–7 (2022) doi:10.1002/acn3.51606.
5. Drange, O. K. *et al.* Genetic overlap between Alzheimer's disease and bipolar disorder implicates the MARK2 and VAC14 genes. *Front. Neurosci.* **13**, 1–11 (2019).
6. Monereo-Sánchez, J. *et al.* Genetic Overlap Between Alzheimer's Disease and Depression Mapped Onto the Brain. *Front. Neurosci.* **15**, 653130 (2021).
7. Desikan, R. S. *et al.* Genetic overlap between Alzheimer's disease and Parkinson's disease at the MAPT locus. *Mol. Psychiatry* **20**, 1588–1595 (2015).
8. Kunkle, B. W. *et al.* Genetic meta-analysis of diagnosed Alzheimer's disease identifies new risk loci and implicates Aβ, tau, immunity and lipid processing. *Nat. Genet.* **51**, 414–430 (2019).
