# Supplementary material for: Local genetic correlations exist among neurodegenerative and neuropsychiatric diseases
Source: NPJ Parkinsons Dis. 2023 Apr 28;9:70. doi: 10.1038/s41531-023-00504-1 (PMC10147945; doi:10.1038/s41531-023-00504-1)
Supplement: Supplementary file 2 — Reporting Summary [file 41531_2023_504_MOESM2_ESM.pdf]

Corresponding author(s): Regina H Reynolds
Mina Ryten

Last updated by author(s): Jan 13, 2023

Reporting Summary

Nature Portfolio wishes to improve the reproducibility of the work that we publish. This form provides structure for consistency and transparency in reporting. For further information on Nature Portfolio policies, see our Editorial Policies and the Editorial Policy Checklist.

Statistics

For all statistical analyses, confirm that the following items are present in the figure legend, table legend, main text, or Methods section.

n/a	Confirmed	
☐	☒	The exact sample size (*n*) for each experimental group/condition, given as a discrete number and unit of measurement
☐	☒	A statement on whether measurements were taken from distinct samples or whether the same sample was measured repeatedly
☐	☒	The statistical test(s) used AND whether they are one- or two-sided<br>*Only common tests should be described solely by name; describe more complex techniques in the Methods section.*
☐	☒	A description of all covariates tested
☐	☒	A description of any assumptions or corrections, such as tests of normality and adjustment for multiple comparisons
☐	☒	A full description of the statistical parameters including central tendency (e.g. means) or other basic estimates (e.g. regression coefficient) AND variation (e.g. standard deviation) or associated estimates of uncertainty (e.g. confidence intervals)
☐	☒	For null hypothesis testing, the test statistic (e.g. F, t, r) with confidence intervals, effect sizes, degrees of freedom and P value noted<br>*Give P values as exact values whenever suitable.*
☒	☐	For Bayesian analysis, information on the choice of priors and Markov chain Monte Carlo settings
☐	☒	For hierarchical and complex designs, identification of the appropriate level for tests and full reporting of outcomes
☐	☒	Estimates of effect sizes (e.g. Cohen's *d*, Pearson's *r*), indicating how they were calculated

Our web collection on statistics for biologists contains articles on many of the points above.

Software and code

Policy information about availability of computer code

Data collection	This study relied on publicly available data, as listed in deposited data in Key resources. The specifics of data collection for each resource can be sourced at their individual websites.
Data analysis	The software utilised in this analysis is outlined in the Software section of Key resources. All code used to process, run genetic correlation analysis and generate figures for the manuscripts is available at https://github.com/RHReynolds/neurodegen-psych-local-corr (doi:10.5281/zenodo.6587707).

For manuscripts utilizing custom algorithms or software that are central to the research but not yet described in published literature, software must be made available to editors and reviewers. We strongly encourage code deposition in a community repository (e.g. GitHub). See the Nature Portfolio guidelines for submitting code & software for further information.

Data

Policy information about availability of data

All manuscripts must include a data availability statement. This statement should provide the following information, where applicable:

- Accession codes, unique identifiers, or web links for publicly available datasets
- A description of any restrictions on data availability
- For clinical datasets or third party data, please ensure that the statement adheres to our policy

Analyses in this study relied on publicly available data, all of which are listed in Key resources. In the case of the PD GWAS without UK Biobank (UKBB) data, summary statistics were kindly provided by the International Parkinson Disease Genomics Consortium: https://pdgenetics.org/.

Human research participants

Policy information about studies involving human research participants and Sex and Gender in Research.

Reporting on sex and gender	In this work utilising publicly available data from genome wide association studies, only summary statistics were utilised with no information regarding underlying individuals. Given this, neither sex nor gender were addressed in this work.
Population characteristics	See above
Recruitment	Participants were not recruited in this study. Information regarding recruitment of participants for individual GWAS studies, and ethics involved, is detailed in each individual GWAS.
Ethics oversight	See above

Note that full information on the approval of the study protocol must also be provided in the manuscript.

Field-specific reporting

Please select the one below that is the best fit for your research. If you are not sure, read the appropriate sections before making your selection.

☐ Life sciences ☒ Behavioural & social sciences ☐ Ecological, evolutionary & environmental sciences

For a reference copy of the document with all sections, see nature.com/documents/nr-reporting-summary-flat.pdf

Behavioural & social sciences study design

All studies must disclose on these points even when the disclosure is negative.

Study description	This study reanalyses publicly available genome wide associations study (GWAS) data (outlined in Key resources) to explore associations of heritability within and between neuropsychiatric and neurodegenerative conditions at loci within the genome.
Research sample	The research explores 3 common and significant neurodegenerative conditions and 3 neuropsychiatric conditions with large, high quality GWAS available.
Sampling strategy	Information regarding sampling, data collection, inclusions and exclusion criteria, and participation can be found in each individual GWAS study.
Data collection	Information regarding sampling, data collection, inclusions and exclusion criteria, and participation can be found in each individual GWAS study.
Timing	Information regarding sampling, data collection, inclusions and exclusion criteria, and participation can be found in each individual GWAS study.
Data exclusions	Information regarding sampling, data collection, inclusions and exclusion criteria, and participation can be found in each individual GWAS study.
Non-participation	Information regarding sampling, data collection, inclusions and exclusion criteria, and participation can be found in each individual GWAS study.
Randomization	Information regarding sampling, data collection, inclusions and exclusion criteria, and participation can be found in each individual GWAS study.

Reporting for specific materials, systems and methods

We require information from authors about some types of materials, experimental systems and methods used in many studies. Here, indicate whether each material, system or method listed is relevant to your study. If you are not sure if a list item applies to your research, read the appropriate section before selecting a response.

Materials & experimental systems

n/a	Involved in the study
☒	☐ Antibodies
☒	☐ Eukaryotic cell lines
☒	☐ Palaeontology and archaeology
☒	☐ Animals and other organisms
☒	☐ Clinical data
☒	☐ Dual use research of concern

Methods

n/a	Involved in the study
☒	☐ ChIP-seq
☒	☐ Flow cytometry
☒	☐ MRI-based neuroimaging
